# Dr. Crawford W. Long

**Published:** 1902-02

**Authors:** Howell Cobb

**Affiliations:** Athens, Ga.


### EDITORIAL NOTES AND COMMENTS.

The Business office of THE JOURNAL-RECORD is No. 64 Marietta Street.

The Editorial office is 318-319-320 The Prudential.

Address all Business communications to Dr. M. B. Hutchins, Mgr.

Make remittances payable to THE ATLANTA JOURNAL-RECORD OF MEDICINE.

On matters pertaining to the Editorial and Original Communications address Dr. Bernard Wolff, Atlanta.

Reprints of original articles will be furnished at cost price. Requests for the same should always be made *on the manuscript.*

We will present, post-paid, on request, to each contributor of an original article, twenty (20) marked copies of THE JOURNAL-RECORD containing such article.

### DR. CRAWFORD W. LONG.

THE following is taken from a daily paper, and contains the facts in relation to the selection of Crawford W. Long to a place in the statuary hall at the national capitol :

*Editor Constitution:*—I wish to present succinctly a few reasons which I think should control the retention of Dr. Crawford W. Long and General James Oglethorpe as Georgia's two representatives in the statuary gallery of famous men to be placed in the capitol at Washington.

My excuse for doing so is this: Dr. Long was my friend and for many years my family physician. Out of this relation grew a personal intimacy which interested me in his claim to be the discoverer of anesthesia and gave me opportunity to investigate fully his claim and that of others to the discovery. Dr. Long's right to this credit can no longer be disputed. But I do not propose to go into this here. Others will present the evidence if it is needed. I desire to call careful consideration to the recognition of this claim by the State of Georgia.

In 1879 the General Assembly of Georgia recognized the right of Dr. Long and chose him as the one whose statue, with that of Oglethorpe, should illustrate Georgia in the Washington gallery of famous men. The resolution of the General Assembly of 1901 does not rescind that of 1879. Oglethorpe and Long were chosen then and the latter's right to be considered the recognized discoverer of anesthesia was investigated, passed upon and finally determined by the General Assembly in 1879.

The resolution of 1901 can only be considered operative so far as now choosing one or more additional names by Georgia. I understand that several of the States have chosen more than two—New York, I believe, has four.

I present with absolute confidence this point: The record is closed as to Oglethorpe and Long. Others may be added, but not to exclusion of these two chosen in 1879. But should the commission raised by resolution of 1901 differ with me on this point, then I urge that in no event should the name of Dr. Long be displaced.

The evidence of Long's right must be conclusive to any fair mind. If so, then I ask, who among all Georgia's sons--distinguished in any field or forum—can compare with Long for this honor?

I had the pleasure of hearing the late Hon. Alexander H. Stephens declare from the rostrum of the University of Georgia that Dr. Crawford W. Long was the undoubted discoverer of anesthesia, and that he was pre-eminent over all other Georgians for this honor. He therefore advised then the choice of Oglethorpe and Long for this statuary hall at Washington.

I need not add testimony of others to that of Stephens. His should be conclusive if we look to the expression of opinion for aid. Would that he were alive to-day to reiterate his choice. He who was so zealous of his own consistency, could he now speak, would urge with all the earnestness of his nature that Georgia, too, be consistent, and in being consistent be just to the memory of her distinguished son, Dr. Crawford W. Long, the discoverer of anesthesia.

Finally, I submit that it would be a deplorable act of injustice for Georgia to set aside the action of the General Assembly of 1879. It is not as though first acting, but in effect to correct the action then taken; hence to deny Dr. Long the right to an honor which has been for years unchallenged.

Europe makes no claim to this discovery, and it is credited there to the United States. In Paris there is a monument to Dr. Long as the discoverer of anesthesia. In Boston, the home of Morton and Johnson, other former claimants, there stands a monument erected to the "discoverer of anesthesia," and for many years this silent witness has emphasized Dr. Long's right. Many years ago the United States Congress asked to recognize Morton and Jackson. Long's claim, with overwhelming evidence of priority, was presented by Senator Dawson, of Georgia, and defeated recognition of these claimants. Long then did not press for Congressional recognition. He was content to submit proof of his right and let it rest. He looked for recognition from his profession. He asked no recognition from the Georgia General Assembly. He died before the action of 1879. Whenever effort was made to prejudice his right he modestly presented evidence of it, and waited spontaneous action, first of his profession and then of the world, to recognize his right. Nor did he ask for pecuniary reward. He desired the honorable fame—not pecuniary reward. He made no concealment of his discovery—applied for no patent, and gave openly and freely to the profession the knowledge of the greatest boon to suffering mankind—painless travail to women, painless surgery to mankind.

Let Georgia stand by her record of 1879; justice to her distinguished son, as well as State pride, demands that Georgia assert the right of Long, and that the State place his statue where all the world will see and know that Georgia proclaims the right of her son. In thus honoring Long the State honors herself.

Yours truly,

Athens, Ga., January 24, 1902.

HOWELL COBB.

If a man's claims to greatness be measured by the benefit which he has bestowed upon his fellow man, there can be no question but that this modest Georgia doctor merits the attribute of the greatest Georgian. His discovery was one that made the world richer and human life sweeter and longer, and the boon to man that proceeded from it is felt wherever in this weary world the hand of civilization has brought the message. Beside the stupendous results of this richest of gifts how empty and whistling are the words of the orator or the oracular utterances of statesmen. Could there be a fuller, purer philanthropy than is practically wrought by Long's discovery? If the prime purpose of life is the pursuit of happiness, can anything conduce more to the attainment of this object than that which abolishes the arch deterrent, pain? It is an instance of the ingratitude of the public that Long's claims should be urged by the medical profession alone, for it is not it that alone reaps the benefit, but that army to whom disease has been robbed of half its horrors by the blessing of anesthesia and whose recruits are gathered from every sort and condition of man. This should ring the praise of Long and urge the primacy of his greatness.

But if the public is callous to the claims of gratitude and duty, every medical man in the State should personally write to some member of the committee and protest against the monstrous injustice, and the black ingratitude that seeks to substitute that of some politician for the great name of Long; some politician whose mouth is stopped with the dust of dead politics and mouldy issues.

